# Health Literacy and Difficulty Accessing Information About the COVID-19 Pandemic Among Parents Who Are Deaf and Hard-of-Hearing

**DOI:** 10.3928/24748307-20221116-01

**Published:** 2022-10

**Authors:** Tyler G. James, Kaila V. T. Helm, Sanjana Ratakonda, Lauren D. Smith, Monika Mitra, Michael M. McKee

## Abstract

People who are deaf and hard-of-hearing (DHH) struggle with information marginalization and limited health literacy, challenging their ability to access information on preventing coronavirus disease 2019 (COVID-19). This study assessed the relationship between language preference, health literacy, and COVID-19 information barriers among parents who are DHH in the United States. Data were drawn from a larger study focused on individuals who are DHH who had given birth in the past 10 years. Respondents completed a web-based survey between March 2020 and July 2021. We segmented respondents by language preference [i.e., American Sign Language (ASL), English, or bilingual ASL/English] and used logistic regression models to test the hypothesis that language preference and health literacy were both associated with COVID-19 information marginalization. Of the total sample (*N* = 417), approximately 17% had limited health literacy, and 22% reported experiencing difficulty accessing information about COVID-19. In adjusted analyses, respondents with limited health literacy ([adjusted odds ratio] aO*R* = 2.245) and Hispanic ethnicity (aO*R* = 2.149) had higher risk of reporting information access barriers. There was no association between language preference and reporting COVID-19 information barriers. However, individuals who are DHH with limited health literacy were at higher risk of experiencing information marginalization during the ongoing COVID-19 pandemic, highlighting the need for tailored information based on access needs. [***HLRP: Health Literacy Research and Practice*. 2022;6(4):e310–e315.**]

Over 49 million people in the United States (approximately 15%) are deaf and hard-of-hearing (DHH) (Agrawal et al., 2008; McKee et al., 2018). State and local governments in the U.S. are underprepared for serving and communicating with DHH communities during emergencies, particularly when sharing essential information (Ivey et al., 2014; National Council on Disability, 2006). The ongoing COVID-19 pandemic has exposed challenges in producing effective and timely health communication (Porat et al., 2020; Schlögl et al., 2021). Understanding how the DHH population can access COVID-19 information is vital to managing the pandemic and achieving U.S. national health objectives to avoid exacerbating inequities among those at risk (De Ver Dye et al., 2021).

One individual-level barrier to effective health communication with DHH people is inadequate health literacy. The DHH population faces widespread information marginalization and limited access to reliable information (Hall et al., 2017), creating significant barriers to accessing effective health communication (McKee et al., 2018, 2020). These barriers place individuals who are DHH at higher risk for delayed access to emergent health messaging and lower funds of health knowledge. Health literacy among the DHH population is often related to language modality. Those who use American Sign Language (ASL) to communicate are nearly 7 times more likely to have inadequate health literacy than English-speakers who are not DHH (McKee et al., 2015). DHH English speakers are also at elevated risk for inadequate health literacy, likely based on reduced access to information, including those that rely on auditory means (Wells et al., 2018).

There is limited research on the relationship between being DHH and experiencing negative consequences related to the COVID-19 pandemic. Early in the pandemic, DHH ASL users sought information about COVID-19 on the internet in English-based text forms (Moreland et al., 2021). This information modality may have presented considerable challenges, particularly when considering inadequate health literacy and limited English proficiency among DHH ASL users (Panko et al., 2021). For example, education status—typically correlated with health literacy—was a significant predictor of knowledge related to the risks of asymptomatic COVID-19 infection (Paludneviciene et al., 2021). These information challenges may have contributed to disparities in COVID-19 infection. In the U.S., individuals who are DHH had higher COVID-19 infection risk than people who are not DHH, with Black, Indigenous, People of Color at the highest risk (De Ver Dye et al., 2021).

Given the increased risk of COVID-19 infection among people who are DHH, the challenges of producing accessible health communication for this population, and overall lack of accessible COVID-related health information (Panko et al., 2021), the purpose of this study was to expand research on assessing inadequate health literacy and COVID-19 information marginalization among DHH people. We hypothesized that language preference (i.e., ASL, English, or bilingual ASL/English) and lower health literacy would be associated with reporting access issues to information about COVID-19.

## Methods

### Data Source and Sample

Data for this study were from the Survey on Pregnancy Experiences of Deaf and Hard-of-Hearing Women collected between May 2020 to July 2021. Participants were recruited through a convenience and purposive sampling strategy across the U.S. through social media, community-based and national organizations, independent living centers, and word-of-mouth. To participate, respondents needed to be DHH with hearing loss occurring before the birth of their most recent child, be age 21 years or older, and self-report birth of a child within the past 10 years. The study procedures were reviewed by the University of Michigan's Institutional Review Board. In total, 620 eligible respondents engaged in the survey.

### Measures

The survey included 80 questions related to respondents' own pregnancy experiences. English survey questions were adapted and translated into ASL and Spanish and placed on a web-based platform. The present study focused on questions related to COVID-19 information access, health literacy, and language preference. Survey covariates included age, racial identity, Hispanic ethnicity, and educational attainment.

The primary outcome variable was measured using a single item, “Have you had trouble getting important and easy access to information about the coronavirus?” Respondents who answered affirmatively were additionally asked, “What challenges getting information about coronavirus have you had?” and were permitted to select multiple closed-ended choices and/or provide free-text, open-ended responses.

Health literacy was measured using a single item, “How confident are you filling out medical forms by yourself?” developed by Chew et al. (2004). This item has strong evidence of validity with scores associated with other health literacy measures (Chew et al., 2004). Respondents could select being from *very confident*, *confident*, *neutral*, *somewhat confident*, or *not confident*. Those indicating being *very confident* or *confident* were coded as having adequate health literacy—all other respondents were coded as having inadequate health literacy.

Language preference was identified by asking, “What language(s) do you prefer to use? Please select all that apply”: (1) sign language or ASL, (2) English, (3) Spanish, or (4) Other (open-ended response). Language preference was recoded into mutually exclusive categories. Specifically, we coded respondents who preferred (1) ASL only, (2) ASL or English, (3) English only, or (4) any other spoken language. Due to the small sample size of the other spoken language group (*n* = 4), this group was removed from the analysis.

### Data Analysis

The analytic sample was comprised of individuals who responded to the primary outcome variable and health literacy item, reducing the sample size to 417. Descriptive statistics were used to describe the sample characteristics and outcome variable distribution. We estimated bivariate relations between language preference and health literacy with experiencing barriers to accessing COVID-19 information using chi-square tests for statistical independence with Cramer's V effect sizes. We then estimated unadjusted and adjusted logistic regressions for the binary outcome of experiencing COVID-19 information accessibility barriers. Logistic regression assumptions and diagnostics were assessed for independence of observations, outliers, linearity of the logit, and multicollinearity. Multi-collinearity was deemed non-problematic [variance inflation factors < 1.0]. Missing data were accounted for using full information maximum likelihood with Monte Carlo integration implemented in Mplus.

## Results

**Figure 1.**
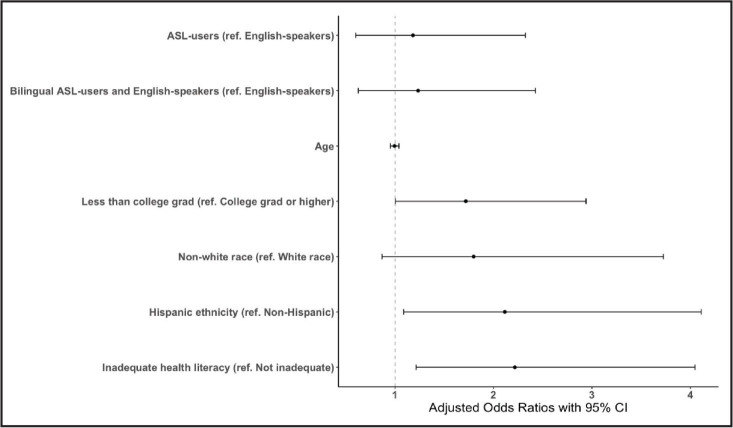
Adjusted odds ratios of logistic regression model assessing the association between language preference and COVID-19 information access barriers among DHH people who experienced pregnancy in the past 10 years. CI = confidence interval; ref. = reference.

The analytic sample was predominantly White (89%) and college-educated (4-year degree or higher, 60%; **Table 1**). Among the 417 respondents who responded to the health literacy and COVID-19 questions, approximately 17% of the sample were identified as having inadequate health literacy, and 22% reported experiencing difficulty accessing information about COVID-19. In bivariate analyses, inadequate health literacy (*p* < .001, Cramer's *V* = 0.21), but not language preference (*p* = .67, Cramer's *V* = 0.04), was associated with COVID-19 information barriers. In un-adjusted models, older respondents, those with lower educational attainment, of Hispanic ethnicity, and those with inadequate health literacy had higher ORs of reporting experiencing COVID-19 information barriers (**Table 2**). When adjusting for covariates, respondents with lower educational attainment, inadequate health literacy, and Hispanic ethnicity had higher risk of experiencing information access barriers (aO*Rs* < 1.7; **Figure 1**).

**Table 1:**
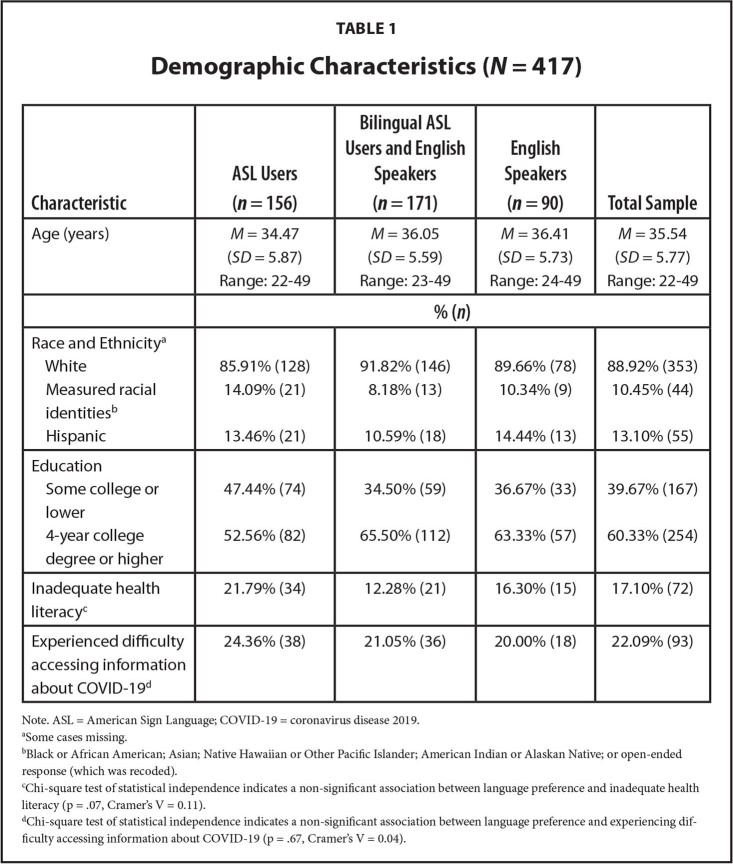
Demographic Characteristics (*N* = 417)

**Characteristic**	**ASL Users (*n* = 156)**	**Bilingual ASL Users and English Speakers (*n* = 171)**	**English Speakers (*n* = 90)**	**Total Sample**

Age (years)	*M* = 34.47	*M* = 36.05	*M* = 36.41	*M* = 35.54
	(*SD* = 5.87)	(*SD* = 5.59)	(*SD* = 5.73)	(*SD* = 5.77)
	Range: 22–49	Range: 23–49	Range: 24–49	Range: 22–49

		**% (*n*)**		

Race and Ethnicity^a^				
White	85.91% (128)	91.82% (146)	89.66% (78)	88.92% (353)
Measured racial identities^b^	14.09% (21)	8.18% (13)	10.34% (9)	10.45% (44)
Hispanic	13.46% (21)	10.59% (18)	14.44% (13)	13.10% (55)

Education				
Some college or lower	47.44% (74)	34.50% (59)	36.67% (33)	39.67% (167)
4-year college degree or higher	52.56% (82)	65.50% (112)	63.33% (57)	60.33% (254)

Inadequate health literacy^c^	21.79% (34)	12.28% (21)	16.30% (15)	17.10% (72)

Experienced difficulty accessing information about COVID-19^d^	24.36% (38)	21.05% (36)	20.00% (18)	22.09% (93)

Note. ASL = American Sign Language; COVID-19 = coronavirus disease 2019.

a Some cases missing.

b Black or African American; Asian; Native Hawaiian or Other Pacific Islander; American Indian or Alaskan Native; or open-ended response (which was recoded).

c Chi-square test of statistical independence indicates a non-significant association between language preference and inadequate health literacy (p = .07, Cramer's V = 0.11).

d Chi-square test of statistical independence indicates a non-significant association between language preference and experiencing difficulty accessing information about COVID-19 (p = .67, Cramer's V = 0.04).

**Table 2:**
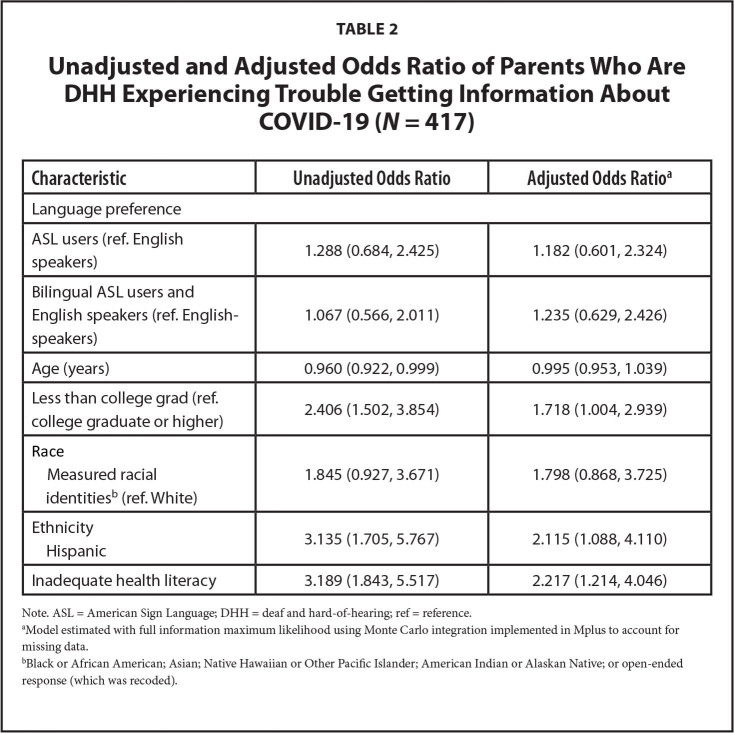
Unadjusted and Adjusted Odds Ratio of Parents Who Are DHH Experiencing Trouble Getting Information About COVID-19 (*N* = 417)

**Characteristic**	**Unadjusted Odds Ratio**	**Adjusted Odds Ratio^a^**

Language preference		

ASL users (ref. English speakers)	1.288 (0.684, 2.425)	1.182 (0.601, 2.324)

Bilingual ASL users and English speakers (ref. English-speakers)	1.067 (0.566, 2.011)	1.235 (0.629, 2.426)

Age (years)	0.960 (0.922, 0.999)	0.995 (0.953, 1.039)

Less than college grad (ref. college graduate or higher)	2.406 (1.502, 3.854)	1.718 (1.004, 2.939)

Race		
Measured racial identities^b^ (ref. White)	1.845 (0.927, 3.671)	1.798 (0.868, 3.725)

Ethnicity		
Hispanic	3.135 (1.705, 5.767)	2.115 (1.088, 4.110)

Inadequate health literacy	3.189 (1.843, 5.517)	2.217 (1.214, 4.046)

Note. ASL = American Sign Language; DHH = deaf and hard-of-hearing; ref = reference.

a Model estimated with full information maximum likelihood using Monte Carlo integration implemented in Mplus to account for missing data.

b Black or African American; Asian; Native Hawaiian or Other Pacific Islander; American Indian or Alaskan Native; or open-ended response (which was recoded).

Additionally, 18 respondents (17 ASL-users) provided open-ended responses describing barriers to retrieving information from public broadcasts about COVID-19, obtaining information about testing and vaccinations, and finding reliable and accessible sources of information.

Here is one response: “I can't find reliable sources [on] the internet. The few I find don't really do a good job of explaining coronavirus to the general public. Almost none of the news sources have reliable, high-quality captioning.”

Finding reliable sources was an issue due to the conflicting information and “click bait” on the internet. In addition, respondents reported a lack of accurate captioning on news stations and issues with ASL interpreters being cut out of the video frame on public emergency broadcasts. An additional barrier was the pace sources at which released new information, with participants reporting that “information [was] conflicting” and that it felt “overwhelming [because of the] changing information every day or every week.”

## Discussion

Although recent research has focused on DHH ASL-users' information seeking behavior with COVID-19 (Moreland et al., 2021; Paludneviciene et al., 2021), little work has focused on the association between health literacy and COVID-19 information marginalization among people who are DHH (Panko et al., 2021). Findings from the present study indicate that language preference is not associated with reporting COVID-19 information access barriers. Instead, results support the hypothesis that people who are DHH with inadequate health literacy, regardless of language preference, remain at higher risk of experiencing COVID-19 information marginalization. In addition, Hispanic respondents who are DHH experienced higher risk of COVID-19 information marginalization. These findings provide potential explanations on the higher COVID-19 infection risk experienced by DHH people. De Ver Dye et al. (2021) established that DHH people in the U.S. were at higher risk for COVID-19 infection, with increased risk observed among minoritized people who are DHH. Potential risk mechanisms can be partially explained by inadequate health literacy and sociodemographic factors (e.g., lower educational attainment), leading to inaccessible health communication about COVID-19 (Panko et al., 2021) and subsequent higher risk of infection. Overall, the proportion of DHH respondents who reported barriers accessing COVID-19–related information was lower than anticipated, likely reflecting information saturation even among this group. However, this may have been an artifact of the recruitment strategy and online data collection. Specifically, our recruitment through social media and community organizations may have overrepresented people who are DHH who had relatively more access to COVID-19 information sources (e.g., those on the web).

## Study Limitations

Due to the larger study's focus, the sample is limited to DHH parents who had given birth in the past 10 years. Due to the sampling design (i.e., convenience sample), these results may not be generalizable to the population of DHH ASL-users and spoken-language users in the U.S. In addition, the outcome variable and health literacy screening items were single-item measures. Future research should use measurement tools with a broader definition of health literacy and gain further information on specific access barriers related to COVID-19 (or future health emergencies). Lastly, due to small cell sizes, we could not test interaction models. Future research should assess the moderating effects of predisposing and enabling factors on information access outcomes.

## Conclusions

People who are DHH with inadequate health literacy are at higher risk of experiencing information marginalization during the ongoing COVID-19 pandemic. Given existing, well-known barriers to individuals' with DHH information access during emergencies, these results further highlight the need for emergency information and health communications to be tailored for accessibility and information needs.
